# Evaluation and Analysis of Mental Health Level of College Students With Financial Difficulties Under the Background of COVID-19

**DOI:** 10.3389/fpsyg.2021.649195

**Published:** 2021-04-09

**Authors:** Yongpeng Feng, Yunting Zhang

**Affiliations:** ^1^School of Design, Jiangnan University, Wuxi, China; ^2^School of Art and Design, Wuxi Vocational Institute of Commerce, Wuxi, China

**Keywords:** COVID-19, college students with financial difficulties, mental health level, all-round development of college students, psychological resilience score

## Abstract

Against the backdrop of COVID-19, the mental health of college students with financial difficulties deserves scientific attention. This paper on the relationship between mental resilience and the mental health of students with financial difficulties summarizes the research on the psychological resilience of students with financial difficulties during the COVID-19 pandemic. It also suggests ways in which to improve the mental health levels of students with financial difficulties by improving their mental resilience.

## Introduction

There is no doubt that COVID-19 has had an influence on both the mental and physical health of students with financial difficulties (hereinafter referred to as SFD) to different degrees. In response to the epidemic prevention and control, colleges are supposed to change with the events, advance with the times, and innovate with the situations, scientifically making effort to ensure the mental health education of college students with financial difficulties. However, under that background, there is still no clear standard to measure their mental health level. Based on questionnaires, introducing the theory of resilience is of research value in order to fully and objectively analyze the mental health of the SFD. Resilience refers to the traits of the individual to recover, repair, or promote health when faced with stress and adversity. These traits with good adaptability will have an impact on individual mental health to various degrees (Shi, 2015). Many studies have shown that the theory of resilience can effectively predict the mental health of the SFD and be conducive to improve the mental health quality of those students.

Generally speaking, scholars home and abroad have made an in-depth study on the mental health level of college students with financial difficulties from the perspective of positive psychology and psychological capital, in order to explore “how it occurs,” “how it evolves,” “how it is evaluated,” and “how it is analyzed” and so on with regards to mental health problems. Foreign countries began to pay attention to the mental health of college students in the 1940s, and in the 1950s mental health education gradually became an important method of social development and individual development of students in various countries. China has not focused on mental health education until the recent decade. Foreign and domestic scholars have done a lot of pioneering research from theoretical analysis and practical research centering on exploring the practical path and optimization of college students' mental health education from the perspective of positive psychology, providing a valuable reference for this study. However, under the background of COVID-19, the above research results are insufficient in the evaluation and analysis of the mental health level of students with financial difficulties, and there is still room for further discussion. For example, there is insufficient attention to explore the causal analysis of the mental health level of the students, the value pursuit of the mental health of the SFD during the COVID-19 pandemic, and the choice of strategy for future development. Most of them focus on elucidating the framework from the theoretical dimension, but less from the practical aspect to set the research field. In addition, part of former research lacks practically problem-orientated and evaluation-orientated analysis and comparative analysis of mental health education of the SFD.

Domestic scholars believe that the continuous improvement of the mental health of the SFD is not only related to the professional growth and future success of college students, but also related to the future and destiny of the country. Because to improve the mental health level of the SFD and promote the all-round development of them is a more effective way to strengthen the fundamental task of “moral education” rather the era mission.

Relevant theoretical foundations include, for example, the exploration of psychological assistance (Zhang, 2013), psychological quality education (Xue and Yan, 2013), and the educational countermeasures of healthy personality (Li and Su, 2019) of the students with financial difficulties from the perspective of positive psychology. Zhang pointed out that from the perspective of positive psychology the psychological assistance of the SFD has returned from focusing on the “negative” problem-oriented model to the “positive” development-oriented model. Cultivating the positive psychology of the SFD needs to develop a positive way of thinking, increase positive psychological experience, and set positive psychological goals. The following aspects should be taken into consideration in order to construct a psychological assistance system for the SFD: construction of a humanized material support system, publicity and education system of positive psychology, psychological assessment and screening system for the SFD, psychological counseling and psychotherapy system, and positive social support system. All these approaches will promote those students to be self-reliant and grow healthily (Zhang and Liao, 2019). According to Li Hua, it is necessary to create a growth environment for the positive emotional experience of the SFD by helping and caring, building a developmental support system for the self-development of the SFD through practical activities, and improving the systematical education system for their all-round development through education and teaching (Li and Su, 2019). Xue Jufeng pointed out that positive psychology emphasizes a more open and appreciative view of human potential, motivation, and ability. As a special group, a few college students with financial difficulties suffer from various kinds of psychological problems under the economic pressure and stress of social competition. Colleges could carry out psychological quality education for these students from the perspective of positive psychology, strengthen positive guidance, and enhance the cultivation of positive emotions. Furthermore, colleges are supposed to encourage them to take the initiative to promote interpersonal communication thus helping them to deal with psychological pressure and nurture the positive psychological quality (Xue and Yan, 2013). Wang Shuzhen explored from three aspects including the significance of positive psychology of the mental health education of the SFD, the current situation and problems of mental health education, and the approach to mental health education. Through the answer to these three aspects, Wang realizes the transformation of the mental health education ideas and educational methods for the SFD and constructs an active and effective mental health education system for them (Wang, 2018). In addition, many scholars conducted research on the mental health level of college students with financial difficulties from the perspective of psychological capital. Relevant theoretical foundations include, for example, the study on mental health status and its corresponding measures (Dong et al., 2016), mental health education (Chen, 2008), and strategies for improving mental health quality (Han, 2019). Liu (2019) explored the factors influencing positive psychological capital of the SFD under the perspective of targeted poverty alleviation. Dong Xiaolei pointed out that psychological capital plays an important role in the mental health and overall development of college students. A comprehensive study of the psychological problems and psychological capital of the SFD can do well to have a more complete and objective understanding of the psychological development of this group. Statistics show that the psychological problems of students with financial difficulties are significantly more than those of students with no financial difficulties (hereinafter referred to as non-SFD). However, their total psychological capital is not only slightly higher than those of students with no financial difficulties, but also has significant advantages in terms of resilience and cooperation. Therefore, colleges' finance assistance work should scientifically grasp the psychological development characteristics of the SFD, shift from “focus on problems” to “focus on advantages,” and strengthen development-oriented aid, thus continuously improving their mental health level by cultivating the group's psychological capital advantages (Dong et al., 2016). Aiming at the psychological problems that college students may encounter during the COVID-19 pandemic, Jin Jie analyzed the factors influencing college students' mental health, and proposed countermeasures from three aspects: strengthening publicity and guidance to improve awareness, establishing psychological counseling and service mechanisms, and developing good living habits, to increase the mental health level of college students (Jin, 2020). Liu Chengyin indicated that due to economic pressure, cultural differences, self-awareness, environment, and other factors, college students with financial difficulties are prone to inferiority, isolation, anxiety, depression, paranoia, social hatred, and other unhealthy psychological phenomena and behaviors. If not given timely attention, it will not only affect their learning and physical and mental health in college, but also influence their work and life in the future when stepping into society, challenging the social harmony and stability. It is of vital importance to study and analyze the causes and manifestations of college students' psychological problems, and to provide targeted assistance and guidance from increasing financial assistance, psychological counseling, creating a sound environment, and enhancing self-adjustment (Liu and Ha, 2018). Liu Ailou pointed out that positive psychological capital is composed of four dimensions: self-efficacy, hope, optimism, and resilience. It is the fundamental driving force for creating a competitive advantage and contributes to individual happiness and success. Nowadays, college students with financial difficulties are facing tremendous life pressures and psychological stress, so we should pay attention to their psychological capital status. Based on the definition of core concepts, the research analyzed the influencing factors of positive psychological capital of the SFD, and proposed strategies to improve their positive psychological capital from the perspective of schools, families, and individuals (Liu, 2019). Under the background of psychological education, Han Zeyu stressed the importance of psychological capital in improving the mental health of the SFD from the perspective of positive psychology, and proposed the development strategy of mental health education and group counseling to cultivate the four core elements of psychological capital (Han, 2019). On the basis of analyzing the manifestations of the mental health problems of the SFD, Chen Guilan explored the reasons behind this from the perspective of psychological capital. She also put forward strategy by increasing their psychological capital to enhance their mental health self-monitoring and development abilities from the four main dimensions of psychological capital, namely self-efficacy, hope, optimism, and resilience (Chen, 2008). Wang Yigui employed the Positive Psychological Capital Questionnaire and the SCL-90 questionnaire to survey 954 local undergraduate college students. The results reveal that: firstly, there are significant differences in psychological capital and mental health among students with financial difficulties in terms of gender, grade, the place of origin, whether they are the only child or not, and other demographic factors. Secondly, there are more psychological problems and lower levels of mental capital of students with financial difficulties. Thirdly, there is a significantly negative correlation between the psychological capital and mental health of students with financial difficulties, which could predict the development of mental health (Wang and Cao, 2019).

Some scholars have also taken the perspective of Happiness Education, Loving Flowers Principle, Peer Psychologically Mutual Supportive Model, helping the impoverished, core self-evaluation, precise psychological assistance, and mental health support system, to evaluate the mental health level of college students with financial difficulties. Relevant theoretical foundations include mental health education of students from families with financial difficulties from the perspective of happiness education (Li and Chen, 2016); enhancing mental health of college students with financial difficulties based on the “Principle of Loving Flowers” (Luo et al., 2010); peer psychologically mutual supportive model—one of the effective ways to improve the mental health level of college students with financial difficulties (Huang, 2015); reflections on the mental health status and precise psychological assistance of family economically disadvantaged students (Ji and Liu, 2019); a survey on the relationship between core self-evaluations and mental health level for college students with financial difficulties (Liu and Jiang, 2019), an investigation and support study on the mental health of students from families with financial difficulties (Wu and Gu, 2018); research on the support systems of psychological healthy for college students from poor families (Wu, 2018), etc. Li Jia pointed out that happiness education involves two aspects: acquiring the ability to obtain happiness and experiencing happiness during the acquiring, and the psychological characteristics of students with financial difficulties contain positive and negative mental states. Happiness education provides the goals and methods for the SFD in the approaches of mental health education and construction of a talent development system, psychological counseling system, and humanistic care system (Li and Chen, 2016). Luo Yuhua indicated that the SFD are an important group of college students, and improving their mental health is a problem that colleges need to pay special attention to. Stick to the loving flowers principle in helping them to get rid of psychological weakness. It can take the aspects of the funding system construction, sound personality, determination sharpening, ability improvement, and campus culture creation to solve the material, mental and ability problems of SFD and improve their mental health dimensionally and coordinately (Luo et al., 2010). According to Huang Chen, the peer psychologically mutual supportive model, as a new form of mental health education, featured homogeneity, convenience, mutuality, and spontaneity. By applying such a model to the psychological assistance work of SFD, it started from meeting the basic psychological needs of SFD, and helped to form a role model, established a harmonious counseling relationship, and embodied the student-oriented approach. Realizing the combination of “helping others and self-help” is one of the new ways to effectively improve the mental health of college students with financial difficulties (Huang, 2015). Under the background of targeted poverty alleviation, Ji Kexin proposed to continuously optimize the funding work for students with financial difficulties. However, a large number of studies have shown that the mental health level of SFD was generally lower than that of non-SFD, thus making psychological assistance another important part of targeted poverty alleviation work. Based on the current situation and reasons analysis, this study put forward countermeasures and prospects of the precise psychological assistance mechanism based on targeted poverty alleviation (Ji and Liu, 2019). Liu Ting mainly investigated the relationship between the “core self-evaluation” and “mental health level” of college students with financial difficulties, explored methods to improve the level of core self-evaluation, and provided a theoretical basis for psychological assistance. In the study, 209 SFD participated in the research by questionnaire and showed that the core self-evaluation was negatively correlated with the total score of the SCL-90 scale and its nine factors. Further regression analysis found that the predictive effect of core self-evaluation on the total score of SCL-90 and its nine factors was statistically significant. It concluded that the core self-evaluation level has a significant impact and predictive effect on the student's mental health level and the higher the core self-evaluation level, the higher the level of mental health (Liu and Jiang, 2019). Based on the stratified sampling, Wu Yingxiong used self-made questionnaires to conduct investigations and found that students' subjective awareness of family economic status had a significant impact on their mental health in their total score of factors. The total scores of factors of students considering their family's economic status as wealthy, upper-middle, average, poor, and extremely poor were 20.5896, 14.8065, 15.6793, 14.8375, and 19.7018, respectively. Among the 10 dimensions describing mental health status, there were significant differences in the scores of students' subjective awareness of family economic status in the seven dimensions of somatization, depression, anxiety, terror, delusion, psychosis, and others, among which the difference in the scores of somatization dimension was hugely significant. It can be seen that the psychological problems of the extremely poor students are relatively prominent, requiring more psychological assistance (Wu and Zhou, 2018). Wu Xudong started from reviewing the literature concerning the psychology of SFD, analyzed the current research status and the existing problems, and then carried out targeted investigations, striving to make a practical overview and exploration on the mental health support system for SFD. Taking a university in Liaoning province as an example and based on the data of college students' psychological and recognition condition, it summarized the behavioral, psychological, and personality characteristics of SFD, and probed into the reasons for their mental health problems. Starting from the “main nature of the mental health support system for SFD,” it answered the questions of “where are the mental health problems of SFD” and “how to establish a mental health support system for them.” After fully and systematically expounding the relevant concepts of the mental health support system for SFD, an attribution analysis of mental health problems of SFD was emphatically discussed with the psychological measurement method with results further empirically elaborated. On the basis of the dialectical relationship between mental health problems and the support systems, an operable mental health support system for SFD was proposed (Wang, 2018).

## Research Methodology

### Theoretic Analysis Framework

The research team selected undergraduates from four universities in Jiangsu Province as the research objects. The samples included students with or without financial difficulties. The evaluation and analysis used the form of questionnaires. The first part of the questionnaire included the basic demographic variables as well as interpersonal relationship and life satisfaction status such as gender, grade, discipline, academic performance, family structure, personality, household registration nature, parent's occupation and educational level, etc. The second part adopts the Connor-Davidson Resilience Scale (CD-RISC), requiring students to choose the level of agreement with the 25 test items, where if some special situations listed in the questionnaire have never happened, they also needed to answer how they will feel according to their experience. In the third part, subjects were asked to answer 90 questions on the five-grade scoring criteria of the SCL-90 scale (“0” stands for the lowest; “10” stands for the highest). The total score is the sum of the 90 items, with 160 points as the clinical boundary, which means that students with more than 160 points may have a psychological disorder. In addition, any factor with a score of more than two is considered positive, indicating that there may be a psychological disorder represented by the factor. If the number of positive factors in each psychological problem is >2, it signifies that there is a problem in this psychological factor.

### Research Settings and Population

In this study, an online questionnaire survey was conducted among four universities with different categories, Jiangnan University, Nanjing University, Nanjing Agricultural University, and Jiangsu Vocational College of Agriculture and Forestry. A total number of 1,218 questionnaires were collected this time, and 1,181 were valid with effective recovery rate of 97%. Data analysis was managed by SPSS for descriptive statistics, independent sample *T*-test, correlation analysis, and analysis of variance.

### Sample Description

According to the results, there are 603 students with financial difficulties, accounting for 51%; there are 577 students without financial difficulties, accounting for 49%. The distribution of demographic variables such as gender, grade, discipline, household registration, parental occupation, and education background is basically comparative for the two types of students. Therefore, here we describe the distribution of the population sample of these demographic variables: Gender: 447 boys, accounting for 38%; 715 girls, accounting for 61%. Grade:367 freshmen, accounting for 31.8%; 615 sophomores, accounting for 52.8%; 119 juniors, accounting for 10%; 62 seniors, accounting for 5.2%; 84 other grades, accounting for 7.1%. Discipline: 118 liberal arts students, accounting for 10%; 511 science and engineering students, accounting for 43.2%; 229 agronomy students, accounting for 19.3%; 186 art students, accounting for 15.7%; 136 other types of students, accounting for 11.5%. Academic performance: 200 students rated as “excellent,” accounting for 16.94%; 494 students rated as “good,” accounting for 41.8%; 456 students rated as “average,” accounting for 38.2%; 30 students rated as “poor,” accounting for 2.5%. Household registration: 480 students as urban residence, accounting for 40.6%; 700 students as rural residence, accounting for 59.2%.

Family structure: among the students with financial difficulties, 108 students are only children, accounting for 17.8%, 496 non-only children, accounting for 82.2%; among the students without financial difficulties, 227 students are only children, accounting for 39.3%, 350 non-only children, accounting for 60.7%. Parent's occupations: among the students with financial difficulties, farming was the most common occupation with 339 parents, accounting for 56.2%; 126 working-class parents, accounting for 20.8%; 17 parents start own business, accounting for 2.8%; 121 unemployed parents, accounting for 20%. Among the students without financial difficulties, working-class parents accounted for 60.3%, with 348 parents ranking the most; followed by 98 farming parents, accounting for 16.9%; then, 50 business entrepreneurs, accounting for 8.6%; 81 other professionals, accounting for 14%. Parental education background: among students with financial difficulties, most parents equipped with junior high school education, accounting for 36.1%, followed by elementary school, accounting for 32.8%, then the third, senior high school education, accounting for 14.4%, and others accounting for 9.4%. Among the students without financial difficulties, most parents equipped with senior high school education, accounting for 25.8%, followed by junior high school, accounting for 25.4%, and the third, bachelor's degree or above, accounting for 22%, and technical secondary school and elementary school accounting for 19.2%. Interpersonal relationship: among the students with financial difficulties, 322 students, accounting for 53.3%, feel that their interpersonal relationship is good and were satisfied; 262 students, accounting for 43.4%, feel their interpersonal relationship is neutral; 19 students, accounting for 3.1%, feel their relationship is poor and are distressed. Among the students without financial difficulties, 333 students feel good and satisfied with their relationship, accounting for 57.1%; 240 students feel their interpersonal relationship is neutral, accounting for 41.55%; four students feel their relationship is poor and are distressed, accounting for 0.6%. Currently facing problems: there is a great difference between SFD and non-SFD. Among the students with financial difficulties, the amount of students facing economic problems is the highest, accounting for 33.8%, followed by learning problems, accounting for 25.5%, and the third, career development problems, accounting for 22.7%, and students with communicative problems and psychological problems account for 6.4% and 3.1%, respectively. Among students without financial difficulties, the number of the students with learning problems is the largest, accounting for 30.8%, followed by career development problem, accounting for 25.9%.

## Data Analysis

### Mental Health Status of SFD

The SCL-90 scores of students with financial difficulties showed no difference in gender, but there are some differences in grade, disciplines, and academic performance (see Table 1). In terms of grade, graduate students scored significantly higher than senior students did (*p* = 0.049 < 0.05), while there was no difference in other grades. It indicated that the mental health level of graduate students was significantly lower than that of senior students. Nevertheless, the sample of graduate students is relatively small in this research, thus having little reference value. In terms of discipline, the scores of science and engineering students were significantly lower than that of agronomy students (*p* = 0.046 < 0.05), while there was no difference in other disciplines, revealing that the mental health level of science and engineering students was significantly higher than that of agronomy students. In terms of academic performance, students labeled as “excellent,” “good,” and “average” were significantly lower than those labeled as “poor” (*p* = 0.000 < 0.05). However, there was no difference between “excellent,” “good,” and “average” students, which signified that the mental health level of students with greater performance (“excellent,” “good,” and “average”) was significantly higher than that of “poor” students. However, since the sample of the students with poor performance is relatively small, this difference remains open to question.

**Table 1:** The SCL-90 scores of SFD.

**Category**	**Group**	**N**	**Mean**	**Std. deviation**	**t**	***P***
Gender	Male	279	142.81	58.290	−0.990	>0.05
	Female	324	147.54	58.643	−0.991	
Grade	Freshman	187	144.9733	53.71034		>0.05
	Sophomore	337	144.2047	60.04896		
	Junior	47	154.4468	62.96576		
	Senior	27	137.5926	57.25399		
	Postgraduate	5	193.6000	79.56947		0.049
Discipline	Liberal Arts	59	147.0678	53.08382		>0.05
	Science and Engineering	304	141.5099	55.81263		
	Agronomy	112	154.4375	65.26078		0.046
	Arts	56	145.7500	44.88743		
	Others	72	145.7500	70.60219		
Performance	Excellent	115	140.9130	53.02094		>0.05
	Good	250	139.9680	52.64131		
	Average	226	149.6106	60.60868		
	Poor	12	220.0000	113.30490		0.000

Different from SCL-90, the psychological resilience of the SFD demonstrated a significant difference in gender, with male students showing significantly higher psychological resilience than female students do (*P* = 0.004 < 0.05) (see Table 2). There was no difference in terms of grades and discipline, but in the perspective of performance, it was different. There were significant differences between “excellent” and “good” students (*p* = 0.003 < 0.05), “good” students and “average” students (*p* = 0.001 < 0.05) in their score of psychological resilience. Whereas, no difference was found between “average” students and “poor” students. It meant that the psychological resilience of students with “excellent” performance was significantly higher than that of students with “good” performance and still, the psychological resilience of “good” students was significantly higher than that of “average” students.

**Table 2:** The psychological resilience scores of SFD.

**Category**	**Group**	**N**	**Mean**	**Std. deviation**	**t**	***P***
Gender	Male	279	89.70	18.922	2.647	0.004
	Female	324	85.98	15.555	2.609	
Grade	Freshman	187	89.29	17.209		>0.05
	Sophomore	337	86.70	17.247		
	Juniors	47	88.45	17.030		
	Senior	27	90.00	16.701		
	Postgraduate	5	76.40	25.076		
Discipline	Liberal Arts	59	88.12	17.300		>0.05
	Science and Engineering	304	88.70	17.611		
	Agronomy	112	85.79	17.992		
	Arts	56	86.55	12.658		
	Others	72	86.99	17.942		
Performance	Excellent	115	94.40	18.601		
	Good	250	88.74	16.054		0.003
	Average	226	83.56	15.890		0.001
	Poor	12	79.83	29.039		>0.05

### Correlation Between Mental Health and Psychological Resilience of SFD

From the correlation analysis, it can be seen that the SCL-90 score and the psychological resilience score of the students with financial difficulties is significantly negative in each dimension and the total score (*p* < 0.01) (see Table 3). When the SCL-90 score is higher, it means a lower mental health level and worse psychological resilience; when the score of SCL-90 is lower, it means a higher mental health level and better mental resilience.

**Table 3:** Correlation between mental health and psychological resilience of SFD.

	**Resilience**	**Strength**	**Optimism**	**Resilience score**
Somatization symptoms	−0.180^**^	−0.261^**^	−0.197^**^	−0.225^**^
Obsessive-compulsive symptoms	−0.241^**^	−0.263^**^	−0.278^**^	−0.272^**^
Interpersonal sensitivity	−0.293^**^	−0.334^**^	−0.291^**^	−0.328^**^
Depression	−0.314^**^	−0.354^**^	−0.326^**^	−0.352^**^
Anxiety	−0.235^**^	−0.296^**^	−0.264^**^	−0.279^**^
Hostility	−0.234^**^	−0.273^**^	−0.224^**^	−0.263^**^
Terror	−0.282^**^	−0.347^**^	−0.273^**^	−0.324^**^
Paranoia	−0.216^**^	−0.291^**^	−0.219^**^	−0.259^**^
Psychotic	−0.243^**^	−0.294^**^	−0.248^**^	−0.279^**^
Others	−0.210^**^	−0.255^**^	−0.227^**^	−0.244^**^
Total score of SCL-90	−0.270^**^	−0.325^**^	−0.284^**^	−0.312^**^

** *Symptom checklist*.

In terms of psychological resilience, strength is the factor most related to mental health level. There are many correlation coefficients with absolute values above 0.3, and other factors have relatively weak influence on the level of mental health. The more confident students are in facing challenges and willing to make the effort, the better their mental health. The less confident and unwilling to make the effort the students are, the worse their mental health.

In terms of mental health level, depression is the most immediately relevant factor to psychological resilience, with all the absolute value of the correlation coefficient reaching 0.3, while other factors deliver a relatively weak impact on psychological resilience. The more positive and happier the students are, the better their psychological resilience is and reversely, the more negative and depressed the students are, the worse their psychological resilience is. In terms of psychological resilience, strength is the most immediately relevant factor to mental health level, with many absolute values of the correlation coefficient reaching 0.3, while other factors deliver a relatively weak impact on mental health level. The more confident students are in facing challenges and willing to make efforts, the better their mental health level is, and reversely, the less confident the students are and the less willing to make efforts, the worse their mental health level is.

### Differences in Mental Health Status Between SFD and Non-SFD

There is a very obvious difference in the scores of SCL-90 between SFD and non-SFD, where except for obsessive-compulsive symptoms (*p* = 0.059 > 0.05), there are significant differences in all other factors and total scores (*p* < 0.05). The scores of most factors of SFD are significantly higher than those of non-SFD. Therefore, the mental health level of SFD is significantly lower than that of non-SFD (see Table 4).

**Table 4:** The SCL-90 scores of SFD and non-SFD.

**SFD**		**N**	**Mean**	**Std. deviation**	**t**	***P***
Somatization symptoms	Yes	603	17.10	7.316	4.851	0.000
	No	577	15.32	5.046	4.889	
Obsessive-compulsive symptoms	Yes	603	19.36	7.498	3.452	0.059
	No	577	17.91	6.865	3.459	
Interpersonal sensitivity	Yes	603	15.61	6.872	3.534	0.000
	No	577	14.28	5.979	3.545	
Depression	Yes	603	22.32	10.125	2.755	0.010
	No	577	20.78	8.999	2.762	
Anxiety	Yes	603	15.93	7.199	3.535	0.000
	No	577	14.59	5.670	3.554	
Hostility	Yes	603	9.32	4.184	3.476	0.000
	No	577	8.55	3.389	3.492	
Terror	Yes	603	10.40	4.661	3.772	0.000
	No	577	9.48	3.552	3.794	
Paranoia	Yes	603	9.19	4.101	3.199	0.000
	No	577	8.49	3.424	3.213	
Psychotic	Yes	603	15.35	6.697	3.859	0.000
	No	577	13.98	5.384	3.878	
Others	Yes	603	10.78	4.773	3.748	0.000
	No	577	9.85	3.631	3.770	
Total score of SCL-90	Yes	603	145.35	58.479	3.932	0.000
	No	577	133.24	46.360	3.951	

There is also a certain difference in the scores of psychological resilience between SFD and non-SFD (see Table 5). The scores of SFD were significantly higher than those of non-SFD in terms of strength (*p* = 0.000 < 0.05) and total score of resilience (*p* = 001 < 0.05). As a result, students with financial difficulties will show more confidence in facing challenges and are more willing to make the effort. In terms of resilience (*p* = 0.060 > 0.05) and optimism (*p* = 0.099 > 0.05), there is no distinct difference, which means that no matter whether have financial difficulties or not, students will not give up easily because of failure but look at the bright side.

**Table 5:** The scores of psychological resilience between SFD and non-SFD.

**SFD**		**N**	**Mean**	**Std. deviation**	**t**	***P***
Resilience	Yes	603	43.57	9.196	−1.016	0.060
	No	577	44.09	8.445	−1.018	
Strength	Yes	603	31.94	6.368	8.054	0.000
	No	577	29.30	4.745	8.105	
Optimism	Yes	603	12.19	2.908	−5.579	0.099
	No	577	13.09	2.627	−5.591	
Resilience score	Yes	603	87.70	17.280	1.311	0.001
	No	577	86.48	14.423	1.317	

Based on the above results, it is not difficult to find out that although the overall level of mental health of SFD is relatively low, their psychological resilience tends not to be at a low level. Their resilience and optimism are equivalent to those of non SFD, and they recover from pressure more easily.

## Discussion

From the above analysis, we can see that the mental health level of SFD is significantly lower than that of non-SFD. However, from the perspective of psychological resilience, SFD are basically the same as non-SFD in terms of resilience and optimism, which is also a concern. Therefore, it is necessary to scientifically master the mental health level and developmental characteristics of SFD as a whole, and help them further improve their psychological tolerance, so as to enhance their ability to solve their own psychological problems.

### Scientifically Prepare for Crisis Identification and Intervention to Achieve Full Coverage

First, adopt flexible and diverse methods. Learn about the mental health status of SFD and find those who might have psychological crisis in time through methods such as individual conversations, class activities, questionnaires, theme education, and other ways among course teachers, counselors, head teachers, tutors, and other groups. Second, focus on the physical and psychological status of scientifically identified SFD. Take the initiative to provide psychological services for students who have experienced psychological distress, psychic trauma, and weak social support systems, or are unwilling to seek help during the COVID-19 pandemic. The study found that graduate students bear more pressure than undergraduates because of scientific research tasks, so the level of mental health of graduate students is lower than that of undergraduates, and the psychological resilience of students with excellent grades is significantly higher than that of students with good grades. Third, give full play to the information linkage of the mental health education network and ensure the dynamic early warning of psychological crisis and the key support for special groups. Actively publicize psychological support hotlines and online counseling services provided by the province and colleges, widely promote psychological self-help methods and channels for help, and solidly carry out individual counseling services in various ways. Fourth, through the establishment of information exchange system, “monthly report” system, psychological counseling, and supervision system, open up special websites for mental health education during the epidemic period, and also establish an online communication platform for psychological counselors. Form the working system of “early detection, early report, early diagnosis, early treatment” to deal with students' mental illness. Fifth, check the mental health status of all students one by one, interview and evaluate the screened potential students, establish mental health records at different levels, and give reasonable intervention to various types and degrees of students with psychological problems.

### Practically Strengthen Humanistic Care for and Pay Attention to Key Groups

First, college counselors should establish psychological files for SFD who have psychological problems during the COVID-19 pandemic with a specific emphasis on psychological counseling and crisis intervention. Meanwhile, give full play to the professional advantages of the school mental health counseling organization, and further provide psychological services of humanistic care for students in need through psychological support hotlines and online psychological counseling. Second, bring into the full play the role of the “five-level” psychological early warning prevention and control system of college, school, grade, class, and dormitory, to carry out psychological self-help for SFD and to resolve their anxiety by spreading positive energy and mutual support. Third, actively realize the popularization of online and offline mental health education through Wechat Official Accounts and themed educational activities, so as to gradually alleviate the psychological panic caused by emotional distress, academic problems, problems on campus, and employment pressure of SFD. Fourth, encourage SFD to take part in psychological salons, psychological sitcoms, and other content-rich and colorful activities. Take Jiangnan University as an example, during the COVID-19 pandemic, the Mental Health Education Center of Jiangnan University formulated the psychological assistance system for epidemic prevention and control. Meanwhile, it actively organized full-time and part-time psychological counselors to actively participate in consultation, supervision, and training based on the full advantages of QQ and telephones. With the help of Wechat Official Accounts such as “Jiangnan University,” “Jiangnan Students,” “Peer Psychological Studio of Jiangnan University” and the website of the Mental Health Education Center, it organized educational activities themed as “the moment we fight with COVID-19,” releasing more than dozens of articles on psychological knowledge of epidemic prevention, such as “Energy of Heart,” “Instruction of Heart,” “Guidance of Heart,” “Share of Heart,” “Care of Heart,” “Growth of Heart” and so on, which are partly originally created to popularize mental health knowledge and promote self-help and mutual assistance, involved in books, courses, music, movies, and other special psychological knowledge of epidemic protection. Based on the above measures, Jiangnan University managed to improve the “psychological immunity” of college students and support the work of epidemic prevention and control.

### Actively Expand and Fully Implement the Channels of Psychological Assistance Resources

First, strengthen economic assistance. The greatest pressure of SFD comes from economic pressure. Therefore, it is necessary to provide material assistance to relieve their psychological pressure caused by the economy. In 2019, 131.689 million yuan was allocated to a total of 48.1759 million college students, an increase of 16.659 billion yuan from the previous year, increasing 14.48%; the national student loan for college students was 34.607 billion yuan, accounting for 16.28% of the total funding. The state's investment in financial aid for SFD has alleviated their psychological pressure to a great extent. Second, improve the channels of psychological assistance. With the full-time teachers of the mental health education center as the important backbone, full-time and part-time counselors, cadres of party and government, head teachers, and tutors as the main force, run mental health education for SFD through the whole process of ideological and political education, combining teacher's guidance with student's peer counseling to achieve all-round education. Third, refine the psychological counseling service system. During COVID-19, the evaluation of the mental health level of SFD should follow the principle of “data-based, value-oriented, and targeted-investigation.” Make full use of the advantages of big data technology, and scientifically build a mental health tracking system for SFD upon their return to school. Combine professional psychological assistance with daily heart-to-heart conversations, and do the utmost in the guidance of students' psychological confusion. Moreover, solve practical difficulties of SFD by providing more internet data usage, protective masks, and other measures in order to really improve the mental health level of SFD.

## Conclusion

In the above research and discussion, the following conclusions are drawn regarding the relationship between the psychological resilience and the mental health level of college students with financial difficulties:

the level of resilience of college students with financial difficulties is good.
there is no significant difference in gender, grade, arts, and science between students with financial difficulties and non-family financial difficulties, but significant difference in academic level. Compared with non-family students with financial difficulties, students with financial difficulties are more confident to face challenges, more willing to work hard, more tenacious, and recover more easily in the face of greater pressure events. It can be seen from the scale that the SCL-90 score and psychological resilience score of students with financial difficulties are significantly negative in each dimension and total score (*P* < 0.01). The higher the score of SCL-90, the lower the level of mental health and the worse the resilience; the lower the score of SCL-90, the higher the level of mental health and the better the resilience.
there is a significant negative correlation between mental health level and resilience level of students with financial difficulties, and resilience level can effectively predict mental health level.

## Data Availability Statement

The raw data supporting the conclusions of this article will be made available by the authors, without undue reservation.

## Ethics Statement

Ethical review and approval was not required for the study on human participants in accordance with the local legislation and institutional requirements. The patients/participants provided their written informed consent to participate in this study.

## Author Contributions

YF conceived the research and drafted the manuscript. YF and YZ jointly completed this article and provided comments on the final version of the manuscript. Both authors contributed to this article and approved the submitted version.

## Conflict of Interest

The authors declare that the research was conducted in the absence of any commercial or financial relationships that could be construed as a potential conflict of interest.

## References

[B1] Chen G. (2008). Discussion on mental health education of impoverished college students from the perspective of psychological capital. China Adult Educ. 38–39.

[B2] Dong X. Liu J. Wang R. Song L. (2016). Study on mental health status of students with financial difficulties and its corresponding measures from the perspective of positive psychology. Stud. Ideol. Educ. 122–124.

[B3] Han Z. (2019). Strategies for improving the mental health quality of college students with financial difficulties from the perspective of psychological capital. Yangtze River Ser. 136–137.

[B4] Huang C. (2015). Peer psychologically mutual supportive model—one of the effective ways to improve the mental health level of college students with financial difficulties. Time Educ. 66–72.

[B5] Ji K. Liu H. (2019). Reflections on the mental health status and precise psychological assistance of family economically disadvantaged students. Psychology 17–19.

[B6] Jin J. (2020). Countermeasures to improve college students' mental health under COVID-19. J. Zhejiang Int. Marit. Coll. 38–40.

[B7] Li H. Su Y. (2019). Study on the educational countermeasures of healthy personality of college students with financial difficulties from the perspective of positive psychology. Stud. Ideol. Educ. 129–130.

[B8] Li J. Chen X. (2016). Mental health education of students from families with financial difficulties from the perspective of happiness education. Manag. Obs. 83–85.

[B9] Liu A. (2019). Influencing factors and coping strategies for the positive psychological capital of impoverished college students from the perspective of targeted poverty alleviation. J. Hubei Norm. Univ. Philos. Soc. Sci. Ed. 151–153.

[B10] Liu C. Ha Z. (2018). Analysis and countermeasures of psychological health problems of students with financial difficulties. Stud. Ideol. Educ. 111–112.

[B11] Liu T. Jiang F. (2019). A Survey on the relationship between core self-evaluations and mental health level for college students with financial difficulties. J. Yueyang Vocat. Tech. Coll. 49–53.

[B12] Luo Y. You M. Huang. (2010). Enhancing mental health of college students with financial difficulties based on “principle of loving flowers.” J. Chongqing Jiaotong Univ. Soc. Sci. Ed. 97–99.

[B13] Shi Y. (2015). Psychological capital status of impoverished college students and improvement measures. Party Build. Ideol. Educ. Sch. 78–79.

[B14] Wang S. (2018). Exploration of mental health education of college students with financial difficulties from the perspective of positive psychology. Educ. Rev. 98–101.

[B15] Wang Y. Cao K. (2019). A study on the mental health of students with financial difficulties from the perspective of psychological capital. J. Chizhou Univ. 83–86.

[B16] Wu X. (2018). The Research on Support System of Psychological Healthy for College Students from Pool Family. Dalian: Dalian University of Technology, 62.

[B17] Wu Y. Zhou H. (2018). Investigation and support study on mental health of students from families with financial difficulties. Guangxi Educ. 10–12.

[B21] Wu Z. Gu J. (2018). A survey on mental health of students from poor families. Guangxi Educ. 10–12.

[B18] Xue J. Yan D. (2013). Psychological quality education for colleges students with financial difficulties from the perspective of positive psychology. Coll. Couns. 62–64.

[B19] Zhang B. Liao S. (2019). Study on psychological assistance to students with financial difficulties from the perspective of positive psychology. Stud. Ideol. Educ. 70–72.

[B20] Zhang L. (2013). Research on the psychological assistance of students with financial difficulties from the perspective of positive psychology. J. Res. Ideological Educ. 70–73.

